# MicroRNA-194: a novel regulator of glucagon-like peptide-1 synthesis in intestinal L cells

**DOI:** 10.1038/s41419-020-03366-0

**Published:** 2021-01-21

**Authors:** Jiao Wang, Di Zhao, Cheng-Zhi Ding, Feng Guo, Li-Na Wu, Feng-Jiao Huang, Yan-Ling Liu, Shui-Ying Zhao, Ying Xin, Sheng-Nan Ma, Hong-Fei Ji, Xiang Wang, Li-Rui Wei

**Affiliations:** 1 Department of Endocrinology, The First Affiliated Hospital of Zhengzhou University, 450052 Zhengzhou, People’s Republic of China; 2 Department of Thoracic Oncology, Henan Provincial Chest Hospital, 450008 Zhengzhou, People’s Republic of China

**Keywords:** Cell biology, Molecular biology

## Abstract

In the status of obesity, the glucagon-like peptide-1 (GLP-1) level usually declines and results in metabolic syndrome. This study aimed to investigate the intracellular mechanism of GLP-1 synthesis in L cells from the perspective of microRNA (miRNA). In the present study, we found that GLP-1 level was down-regulated in the plasma and ileum tissues of obese mice, while the ileac miR-194 expression was up-regulated. In vitro experiments indicated that miR-194 overexpression down-regulated GLP-1 level, mRNA levels of proglucagon gene (*gcg*) and prohormone convertase 1/3 gene (*pcsk1*), and the nuclear protein level of beta-catenin (β-catenin). Further investigation confirmed that β-catenin could promote *gcg* transcription through binding to transcription factor 7-like 2 (TCF7L2). miR-194 suppressed *gcg* mRNA level via negatively regulating TCF7L2 expression. What’s more, forkhead box a1 (Foxa1) could bind to the promoter of *pcsk1* and enhanced its transcription. miR-194 suppressed *pcsk1* transcription through targeting *Foxa1*. Besides, the interference of miR-194 reduced palmitate (PA)-induced cell apoptosis and the anti-apoptosis effect of miR-194 inhibitor was abolished by TCF7L2 knockdown. Finally, in HFD-induced obese mice, the silence of miR-194 significantly elevated GLP-1 level and improved the metabolic symptoms caused by GLP-1 deficiency. To sum up, our study found that miR-194 suppressed GLP-1 synthesis in L cells via inhibiting TCF7L2-mediated *gcg* transcription and Foxa1-mediated *pcsk1* transcription. Meanwhile, miR-194 took part in the PA-induced apoptosis of L cells.

## Introduction

Over the last several decades, obesity has become a global epidemic due to overeating and lack of exercise. According to statistics, about 700 million people in the world are suffered from obesity^1^. Generally, obesity is accompanied by metabolic disturbances (such as dyslipidemia, hyperglycemia, and hypertension) and obese individuals are more prone to cancer and heart disease^2^. Although lifestyle interventions, involving diet and physical activity, are cornerstones for obesity management, the use of pharmaceutical agents is indispensable for the long-term treatment of obesity.

Glucagon-like peptide 1 (GLP-1), a gut hormone mainly secreted by intestinal L cells, has been reported to be an essential regulator in glucose metabolism via stimulating insulin secretion and inhibiting glucagon release^3^. Since GLP-1 could effectively improve insulin resistance, induce satiety, and suppress appetite, it is considered as a promising hormone for treating metabolic syndrome caused by obesity^4^. There are two essential participators in the progress of GLP-1 production, proglucagon gene (*gcg*) and prohormone convertase 1/3 (PC1/3). Under normal conditions, in intestinal L cells, *gcg* codes for proglucagon, then, the proglucagon is hydrolyzed to GLP-1 in the presence of PC1/3^5^. However, under the stimulation of a high-fat diet (HFD), the intestinal L cell function was impaired and the mRNA levels of *gcg* and *pcsk1* (the gene of PC1/3) were down-regulated, resulting in the defective GLP-1 production^6–8^. However, the specific mechanism of HFD impairing the GLP-1 production which was mediated by *gcg* and PC1/3 remains perplexing.

MicroRNA (miRNA) is a class of 19-24 nucleotides non-coding RNA and its dysregulation has been observed in the occurrence of obesity^9^. Olivo-Marston et al.^10^ screened out eighteen miRNAs which were abnormally expressed in the colons of HFD-induced obese mice utilizing microarray, among them, the miR-194 level was significantly increased. In addition, the high expression of miR-194 aggravated cardiac injury and mitochondrial dysfunction in obese mice^11^. The interference of miR-194 favored the recovery of dietary-induced non-alcoholic fatty liver disease by reducing the inflammatory response^12^. The above researches indicated that miR-194 has been implicated to play a role in obesity, while whether miR-194 affects GLP-1 production during obesity is still unknown.

It is known that obese individuals frequently exhibit elevated levels of interleukin 6 (IL-6) and free fatty acid (FFA)^13,14^. As an enteroendocrine cell mainly located in the distal ileum and colon, L cells can sense the cytokines and luminal nutrients, and transform these pieces of information into stimulation of GLP-1 secretion^15^. As reported, in the obese mice, the plasma IL-6 level was elevated and the high level of IL-6 increased GLP-1 synthesis and secretion in intestinal L cells via promoting *gcg* transcription^5^. Meanwhile, as the most abundant saturated FFA, palmitate (PA) was cumulated in the large intestine of obese mice and the PA treatment contributed to the apoptosis of intestinal L cells in vitro^16^. The above data hinting that L cells could be affected by IL-6 and PA during obesity. What’s more, previous studies reported that miR-194 was down-regulated by IL-6 in nucleus pulposus cells^17^ while up-regulated by PA in hepatoma cell line HepG2^12^. Inspired by the previous studies, we speculated that miR-194 might be involved in the regulatory effect of IL-6 and PA on GLP-1 synthesis during obesity.

Herein in this study, we investigated the specific modulatory mechanisms of miR-194 on the secretion of GLP-1 in intestinal L cells under the HFD stimulation, hoping to provide new intervention targets for the deficiency of GLP-1 secretion in obesity.

## Materials and methods

### Animal experiments

Six-week-old male C57BL/6 mice (*n* = 30, Shanghai SLAC Laboratory Animal Co., Ltd, China) were divided into two groups. In the control group (*n* = 15), mice were fed with a normal chow diet (D12450H, Research Diets, USA). In the HFD group (*n* = 15), mice were fed with a 60% high-fat diet (D12492, Research Diets, USA). Twelve weeks later, the feces of mice were collected and the contents of PA were detected using chromatography. Blood samples were obtained for the measurement of total cholesterol (TG), triglyceride (TC), glucose, and IL-6 levels using an automatic biochemistry analyzer (BECKMAN, USA) or a Mouse IL-6 ELISA Kit (Abcam, UK), respectively. Adipose, pancreatic, and ileum tissues were collected for the follow-up experiments.

To examine the effect of miR-194 inhibition on HFD mice, six-week-old male C57BL/6 mice were divided into four groups: control (*n* = 6), HFD (*n* = 6), HFD + miR-194 antagomir (*n* = 6), and HFD + negative control of miR-194 antagomir (antagomir NC, *n* = 6). In HFD + miR-194 antagomir and HFD + antagomir NC group, after 8 weeks of HFD, mice were treated with miR-194 antagomir or antagomir NC as previously described^18^. Briefly, mice were anesthetized using pentobarbital sodium (50 mg/kg). Then, intracolonic enemas were performed using a 3.5 cm long polyethylene cannula (Intramedic PE-20 tubing, Becton Dickinson, USA) attached to an insulin syringe and introduced into the colon reaching ~3 cm from the anus. miR-194 antagomir or antagomir NC (10 mg/kg) was instilled into the colon using a 1 ml syringe. Mice were then returned to the cages and continued to be fed with HFD. The intracolonic enemas were performed once a week. Four weeks later, the mice were sacrificed. Blood samples were obtained for the measurement of TG, TC, and IL-6 levels. Adipose, pancreatic, ileum, and colon tissues were collected for the follow-up experiments. The animal was chosen and group-dividing randomly and the investigators were blinded to the group allocation. All animal procedures were in accordance with institutional guidelines and approved by the Ethics Committee of the First Affiliated Hospital of Zhengzhou University.

### Cell culture and transfection

An L-like cell line, STC-1, were purchased from Cell Bank of Type Culture Collection of the Shanghai Institute of Cell Biology (China), authenticated and tested for mycoplasma contamination from the Procell (China), and maintained in Dulbecco’s modified eagle medium (DMEM; ATCC, USA) containing 10% fetal bovine serum (GIBCO, USA) with 5% CO_2_ at 37°C.

RNAi vector (miR-194 inhibitor, si-ctnnb1, si-pcsk1, si-TCF7L2, and si-Foxa1), over-expression vectors (miR-194 mimic) and relative negative controls (NC, si-NC, and miRNC) were synthesized by RIBOBIO (China). For transfection, cells were seeded in 6-well plates with a concentration of 4 × 10^5^ cells/well. When the cells were cultured to 70% confluence, cells were incubated with 2 ml Opti-MEM medium (GIBCO, USA) containing plasmids (1 μg) and Lipofectamine 3000 (2.5 μl; Invitrogen, USA). Six hours later, the original medium was replaced with 2 ml fresh DMEM complete medium. Eighteen hours later, cells were incubated with different concentrations of IL-6 or PA for another 24 h. The sequences of the transfected components were shown in Table 1.

**Table 1:** Sequences of the transfected components used in the experiments.

Transfected components	Sequences
Si-NC	5′-UUCUCCGAACGUGUCACGUCU-3′
Si-TCF7L2	5′-CGAAAGUUUCCGAGAUAAAUC-3′
Si-Ctnnb1	5′-GGGUGCUAUUCCACGACUAGU-3′
Si-Pcsk1	5′-CAGUGACUAUGUUGAUGUAUU-3′
Si-Foxa1	5′-AGCACAAGCUGGACUUCAAGG-3′
miR-194 inhibitor	5′-TCCACATGGAGTTGCTGTTACA-3′
NC	5′-AGGUTCAACUTGACGTACAGGA-3′
miR-194 mimic	5′-UGCAGCAGCUUCTGCATGTCCT-3′
miRNC	5′-ACATTGTCGTTGAGGTACACCT-3′

### Quantitative real-time PCR (qRT-PCR)

Total RNA was extracted from cells and tissues of mice using TRIZOL reagent (Invitrogen, USA). The concentration and purity of RNA were measured by the ultraviolet spectrophotometer. Reverse transcription was conducted for the synthesis of cDNA using the SuperScript IV First-Strand Synthesis System (Invitrogen, USA) or miScript Reverse Transcription kit (QIAGEN, Germany). The qRT-PCR assay was conducted using the Platinum Quantitative RT-PCR ThermoScript One-Step Kit (Invitrogen, USA) or miScript SYBR Green PCR kit (QIAGEN, Germany) in ABI 7500 Real-Time PCR System (Applied Biosystems, CA, USA). U6 or GAPDH was used as the endogenous control. The sequences of qRT-PCR primers were shown in Table 2.

**Table 2:** Sequences of the primers used in the experiments.

qRT-PCR Primers	Sequences
*miR-194*	RT-Primer 5′-GTCGTATCCAGTGCAGGGTCCGAGGTATTCGCACTGGATACGACTCCACA-3′
	F: 5′-CGCGTGTAACAGCAACTCCA-3′
	R: 5′-AGTGCAGGGTCCGAGGTATT-3′
*gcg*	F: 5′-CGTGCCCAAGATTTTGTGCA-3′
	R: 5′-CCCTTCAGCATGCCTCTCAA-3′
*pcsk1*	F: 5′-GCTCCATCTTTGTCTGGGCT-3′
	R: 5′-ACTGCTGTAGGAGGTAGCCA-3′
*Sox17*	F: 5′-GCTCCAGTCTCGGACTATGC-3′
	R: 5′-CCGTAGTACAGGTGCAGAGC-3′
*Chd8*	F: 5′-AAGCCCAGGTAACTCAAC-3′
	R: 5’-TTCACATCGTCGGCGTCT-3′
*U6*	F: 5′-CTCGCTTCGGCAGCACA-3′
	R: 5′-AACGCTTCACGAATTTGCGT-3′
*GAPDH*	F: 5′-TCACTCAAGATTGTCAGCAA-3′
	R: 5′-AGATCCACGACGGACACATT-3′
**3**′**UTR primers**	**Sequences**
*TC7F2*	F: 5′-CGAGCTCcccccttgacctcctagtca(SacI)-3′
	R: 5′-GCTCTAGAtgctttctggacagtctgct(XbaI)-3′
*Foxa1*	F: 5′-CGAGCTCacacagacacacacacacca(SacI)-3′
	R: 5′-GCTCTAGAagggagaaaggggaggaaga(XbaI)-3′
**Promoter primers**	**Sequences**
*gcg*	F: 5′-CGAGCTCacatgcctaccactacccct(SacI)-3′
	R: 5′-CGGCTAGCtctgcaccagggtgctgtgc(NheI)-3′
*pcsk1*	-2.2k F: 5′-CGAGCTCctattccaaaacaccttctgc(SacI)-3′
	-1.5k F: 5′-CGAGCTCgcccaggttcagtgagagac(SacI)-3′
	-0.8k F: 5′-CGAGCTCgcacagcttcaagtcagtgc(SacI)-3′
	R: 5′-CGGCTAGCaatgagtgtttacacgtca(NheI)-3′

### Western blot

Western blot was performed as previously described^19^. Whole-cell protein samples were purified using RIPA lysis buffer (Cwbio, China) and the nuclear protein samples were purified using a Nucleoprotein Extraction Kit (Sangon Biotech, China). The primary antibodies used in the experiment were as follows: anti-PC1/3 (1:1000), anti-beta-catenin (β-catenin; 1:5000), anti-transcription factor 7-like 2 (TCF7L2; 1:25000), anti-forkhead box a1 (Foxa1; 1:1000), anti-cleaved-caspase 3 (c-caspase 3; 1:500), anti-β-actin (1:5000), and anti-Lamin B1 (0.1 µg/ml) (all purchased from Abcam, UK).The secondary antibody used in the experiment was Goat Anti-Rabbit IgG H&L (1:5000; Abcam, UK).

### Measurements of GLP-1 in plasma and culture medium

Plasma samples of mice were collected after anesthesia in the presence of aprotinin (2 μg/ml), EDTA (1 mg/ml), and diprotin (0.1 mM). GLP-1 (active) level was measured using a Mouse GLP-1 Elisa Kit (Solarbio, China) according to the manufacturer’s instruction.

Cell culture medium was collected in the presence of halt protease and phosphatase inhibitor cocktail (10 μl/ml, to suppress the degradation of GLP-1; Thermo Fisher Scientific, USA).

### Dual-luciferase reporter assay

To verify the combination between miR-194 and *TCF7L2*, the sequence of *TCF7L2* 3’UTR was amplified and inserted into a pmirGLO vector (Promega, USA). 0.5 μg plasmid containing *TCF7L2* 3′UTR and 20 nM miR-194 mimic/miRNC were cotransfected into well-grown STC-1 cells by using lipofectamine 2000 (ThermoFisher, USA). Forty-eight hours after transfection, cells were lysed and the luciferase activity was measured by dual-luciferase reporter assay system (Promega, USA). To verify the combination between miR-194 and *Foxa1*, the luciferase activity of *Foxa1* 3′UTR was measured in the same way. The primer sequences for amplifying *TCF7L2*/ *Foxa1* 3′UTR were shown in Table 2.

To measured the prompter activity of *gcg*, the sequence of *gcg* promoter was amplified and inserted into pGL3-basic plasmids (Promega, USA). Cells were then cotransfected with pGL3-basic plasmid containing *gcg* promoter + si-TCF7L2 + pcDNA-β-catenin/ pcDNA empty vector using lipofectamine 2000 (ThermoFisher, USA). Forty-eight hours later, the luciferase activity was measured by the dual-luciferase reporter assay system (Promega, USA). To verify the promoter activity of *pcsk1*, the luciferase activity of the *pcsk1* promoter was measured in the same way. The primer sequences for amplifying *gcg*/*pcsk1* promoters were shown in Table 2.

### Co-immunoprecipitation (Co-IP)

Co-IP was performed as previously described^20^. Briefly, protein lysates from 4 × 10^5^ STC-1 cells which were transfected with miR-194 inhibitor or NC were incubated with anti-TCF7L2 antibody (Abcam, USA). Twelve hours later, cells were incubated with Protein A/G PLUS agarose resin (Yeasen, China) for 6 h. Then, the agarose resin was extensively washed with washing buffer and centrifuged. Proteins in the immunocomplexes were extracted in SDS sample buffer and used for western blot to identify the interaction between TCF7L2 and β-catenin.

### Chromatin immunoprecipitation (CHIP)

The combination of Foxa1 and *pcsk1* promoter was assessed using the ChIP-IT Kit (Active Motif, USA) according to the manufacturer’s instructions. Briefly, anti-Foxa1 antibody-coated beads were used to pull down the Foxa1 complexes from 1 × 10^7^ STC-1 cells. The beads were washed three times with washing buffer. The beads were then eluted and subjected to reverse crosslinking. Then, the complex was assessed by qRT-PCR. For qRT-PCR, the primer of the *pcsk1* promoter was synthesized by RIBOBIO (China). The sequences of *pcsk1* promoters: F 5’-TACACAAACACACGTGTCCG-3’; R 5’-CCTAAAGGGAGTGGGAGTGG-3’.

### Cell viability assay

The cell viability was assessed by methyl thiazolyl tetrazolium (MTT) assay. Cells were seeded in 6-well plates with a concentration of 4 × 10^5^ cells/well. When the cells were cultured to 70% confluence, the cells of each well were incubated with 10 μl MTT (Cwbio, China) at 37 °C in the dark. Four hours later, the supernatant was removed and formazan crystals were dissolved by the addition of dimethyl sulfoxide (DMSO, 150 µl/well; Cwbio, China) at 37 °C for 15 min. The absorbance intensity was measured at 490 nm. Cell viability (%) = (mean absorbance in test wells)/(mean absorbance in control wells) ×100.

### Cell apoptosis assay

Cells were seeded in 6-well plates at 4 × 10^5^ cells/well. When the cells were cultured to 70% confluence, cells were collected and washed with PBS. Cell apoptosis was measured using the Annexin V-FITC Apoptosis Detection Kit (Univ-bio, China). In brief, cells were resuspended in 195 μl Annexin V-EGFP Binding Buffer and incubated with 5 μl Annexin V-FITC for 15 min at room temperature in the dark. Then, 10 μl Propidium Iodide Staining Solution was added to the cells. Five minutes later, cell apoptosis was measured by the flow cytometer (BD, USA) immediately.

### Oral glucose tolerance test (OGTT) and insulin tolerance test (ITT)

After feeding with a normal chow diet or HFD for 12 weeks, mice received OGTT and ITT. For OGTT, after a 12-hour fast, mice were gavaged with glucose (3 g/kg body weight). For ITT, after a 4-hour fast, mice received an intraperitoneal injection of insulin (0.75 U/kg). Blood samples were taken from the tail vein at 0, 15, 30, 60, 90, and 120 min after administration of glucose or insulin. Plasma glucose levels were measured by Glucotrend (Roche, GER) and plasma insulin levels were measured using the Insulin Mouse ELISA Kit (Invitrogen, USA).

### H&E staining

Adipose and pancreatic tissues were fixed in 4% formaldehyde, embedded with paraffin and sectioned, following hematoxylin and eosin staining. The sections were then visualized under the microscope (Nikon, USA) and the areas of adipocytes were analyzed using Image Pro-Plus.

### Immunohistochemistry

Ileum and colon tissues were fixed in 4% formaldehyde, embedded with paraffin, and sectioned. Antigen retrieval was performed using proteinase K (Solarbio, China) and endogenous peroxidase activity was deactivated using 3% H_2_O_2_. Then, sections were blocked with 5% bovine serum albumin (BSA; Solarbio, China) and incubated with primary antibody against GLP-1 (1:500; Abcam, UK) overnight. Then, sections were incubated with the secondary antibody and ABC solution (Solarbio, China). After the diaminobenzidine solution and hematoxylin staining, sections were mounted and visualized under the microscope (Nikon, USA). The GLP-1 positive cells per high-power field (HPF) were calculated in 10 random HPFs.

### Statistical analysis

SPSS 18.0 software was used for data analysis. Measurement data were expressed as mean±standard deviation (SD). Data were analyzed by student’s *t*-test (for two experimental groups) or one-way ANOVA (for multiple experimental groups). The significance was set at *P* < 0.05.

## Results

### miR-194 was up-regulated in HFD-induced obese mice

**Fig. 1:**
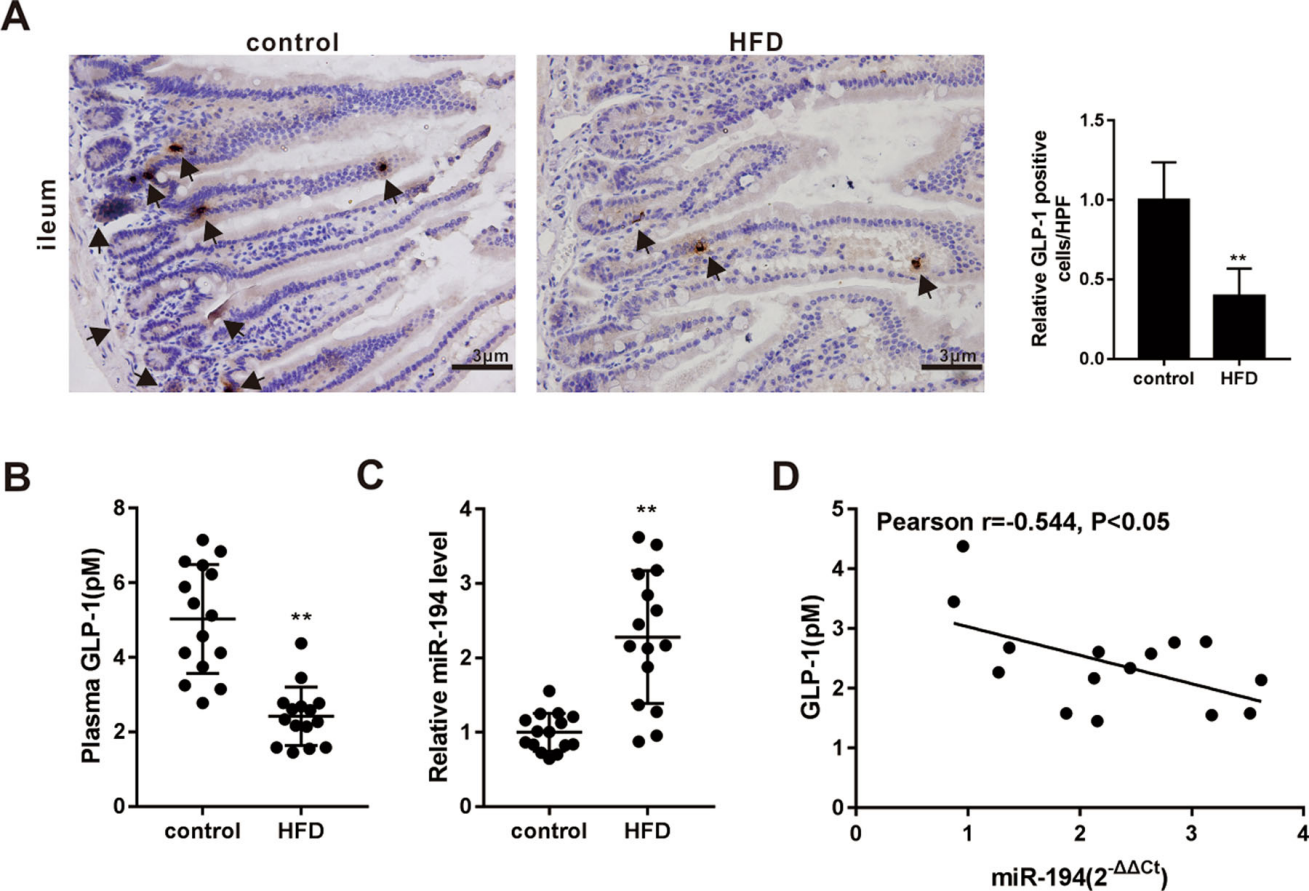
miR-194 was up-regulated in HFD-induced obese mice. Male C57BL/6 mice were fed with a normal chow diet (control group, *n* = 15) or high-fat diet (HFD group, *n* = 15) for 12 weeks. **a** Representative immunohistochemical staining for glucagon-like peptide-1 (GLP-1) in ileum tissues (scale bar=3 μm) and quantified results were expressed as the mean numbers of GLP-1-positive cells in 10 high-power fields (HPFs). **b** The plasma level of active GLP-1 was assayed by ELISA. **c** The miR-194 expression in ileum tissues was measured by qRT-PCR. **d** Pearson correlation analysis between the plasma level of GLP-1 and ileac miR-194 expression. **P < 0.01 vs control.

C57BL/6 mice were fed with a normal chow diet or HFD for 12 weeks. As shown in Supplemental Fig. 1a–d, compared with the control group, the HFD group displayed an apparent weight gain, higher plasma levels of TG, TC, and glucose, and increased areas of adipocyte and islet, confirming that HFD successfully induced the obesity in mice. Moreover, in response to HFD, the GLP-1 expression was down-regulated in both ileum tissues and plasma samples (Fig. 1a and b) while the ileac miR-194 expression was up-regulated (Fig. 1c). Then, the further Pearson correlation analysis demonstrated a negative correlation (*r* = −0.544, *P* < 0.05) between plasma GLP-1 level and ileac miR-194 expression (Fig. 1d).

### miR-194 overexpression suppressed the GLP-1 synthesis in an L-like cell line

**Fig. 2:**
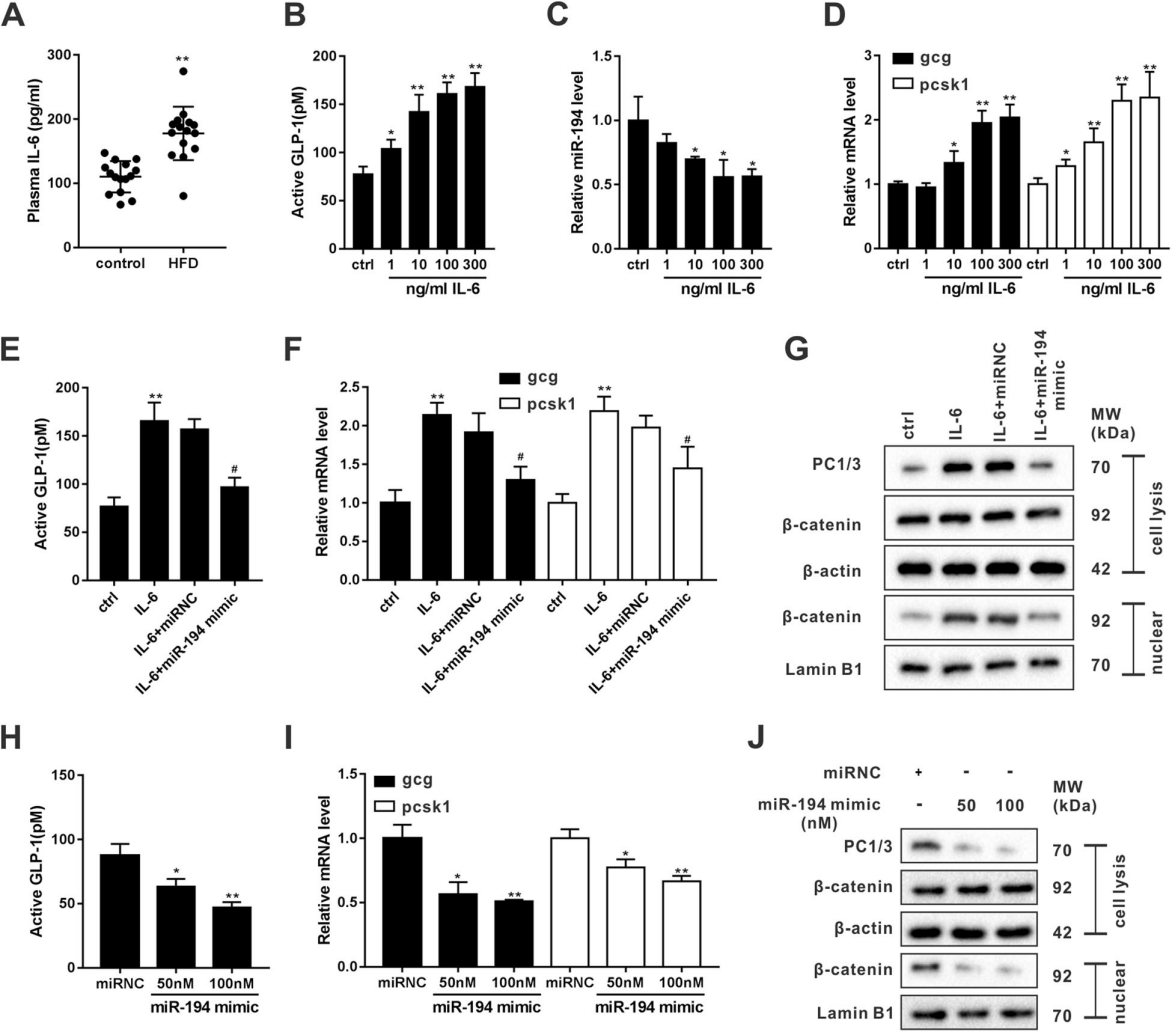
miR-194 overexpression suppressed GLP-1 level in an L-like cell line. **a** The plasma levels of IL-6 in the control and HFD groups were measured by ELISA. **b**–**d** The STC-1 cells were incubated with different concentrations of IL-6 (0, 1, 10, 100, and 300 ng/ml) for 24 h. ctrl=control. **b** The active GLP-1 level in the supernatant was measured by ELISA. **c** The miR-194 expression was measured by qRT-PCR. **d** The mRNA levels of *gcg* and *pcsk1* were measured by qRT-PCR. **e**–**g** The STC-1 cells were transfected with miR-194 mimic (50 nM) or its negative control (miRNC). Twenty-four hours later, cells were then incubated with IL-6 for 24 h. **e** The active GLP-1 level in the supernatant was measured by ELISA. **f** The mRNA levels of *gcg* and *pcsk1* were measured by qRT-PCR. **g** The protein levels of PC1/3, whole-cell β-catenin, and nuclear β-catenin were determined by western blot. β-actin and Lamin B1 were used as loading controls for cell lysis and nuclear, respectively. **h**–**j** The STC-1 cells were transfected with miR-194 mimic (50 nM or 100 nM) or miRNC. Forty-eight hours after transfection, the (**h**) active GLP-1 levels, (**i**) mRNA levels of *gcg* and *pcsk1*, and (**j**) protein levels of PC1/3, whole-cell β-catenin, and nuclear β-catenin were measured. *P < 0.05, **P < 0.01 vs control or miRNC. ^#^*P* < 0.05 vs IL-6+miRNC.

As shown in Fig. 2a, the HFD group exhibited a significantly higher plasma level of IL-6 than that of the control group. Meanwhile, the results of in vitro experiments showed that IL-6 could elevate GLP-1 level and the mRNA levels of *gcg* and *pcsk1* (two essential genes relevant to GLP-1 production^5^) in a dose-dependent way (Fig. 2b and d), confirming that IL-6 was a promoter for GLP-1 synthesis. Besides, the results of Fig. 2c showed that IL-6 negatively regulated the miR-194 expression. Further experiments depicted that miR-194 overexpression could reduce GLP-1 level, suppress transcriptions of *gcg* and *pcsk1*, and inhibit PC1/3 expression in untreated STC-1 cells (Fig. 2h–j), validated that miR-194 was a regulator of GLP-1 production in intestinal L cells. To explore whether IL-6 promoting GLP-1 synthesis via suppressing miR-194 expression, we overexpressed miR-194 in STC-1 cells utilizing miR-194 mimic (50 nM). Interestingly, in IL-6-treated STC-1 cells, the overexpression of miR-194 reduced the IL-6-induced GLP-1 secretion (Fig. 2e), abolished the promoting effect of IL-6 on the mRNA levels of *gcg* and *pcsk1* and the protein level of PC1/3 (Fig. 2f and g). The previous study showed that the transcriptional activation of *gcg* could be promoted by Wnt/β-catenin signaling pathway^21^. Indeed, the nuclear expression of β-catenin was accumulated (the marker of Wnt/β-catenin signaling activation^22^) in IL-6-treated cells, while the overexpression of miR-194 abrogated the effect of IL-6 (Fig. 2g). Similarly, in miR-194-overexpressed STC-1 cells, the nuclear expression of β-catenin was reduced (Fig. 2j). These data indicating that the Wnt/β-catenin signaling was involved in the regulatory effect of IL-6/miR-194 on *gcg* transcription.

### miR-194 reduced GLP-1 synthesis via repressing TCF7L2-mediated gcg transcription

**Fig. 3:**
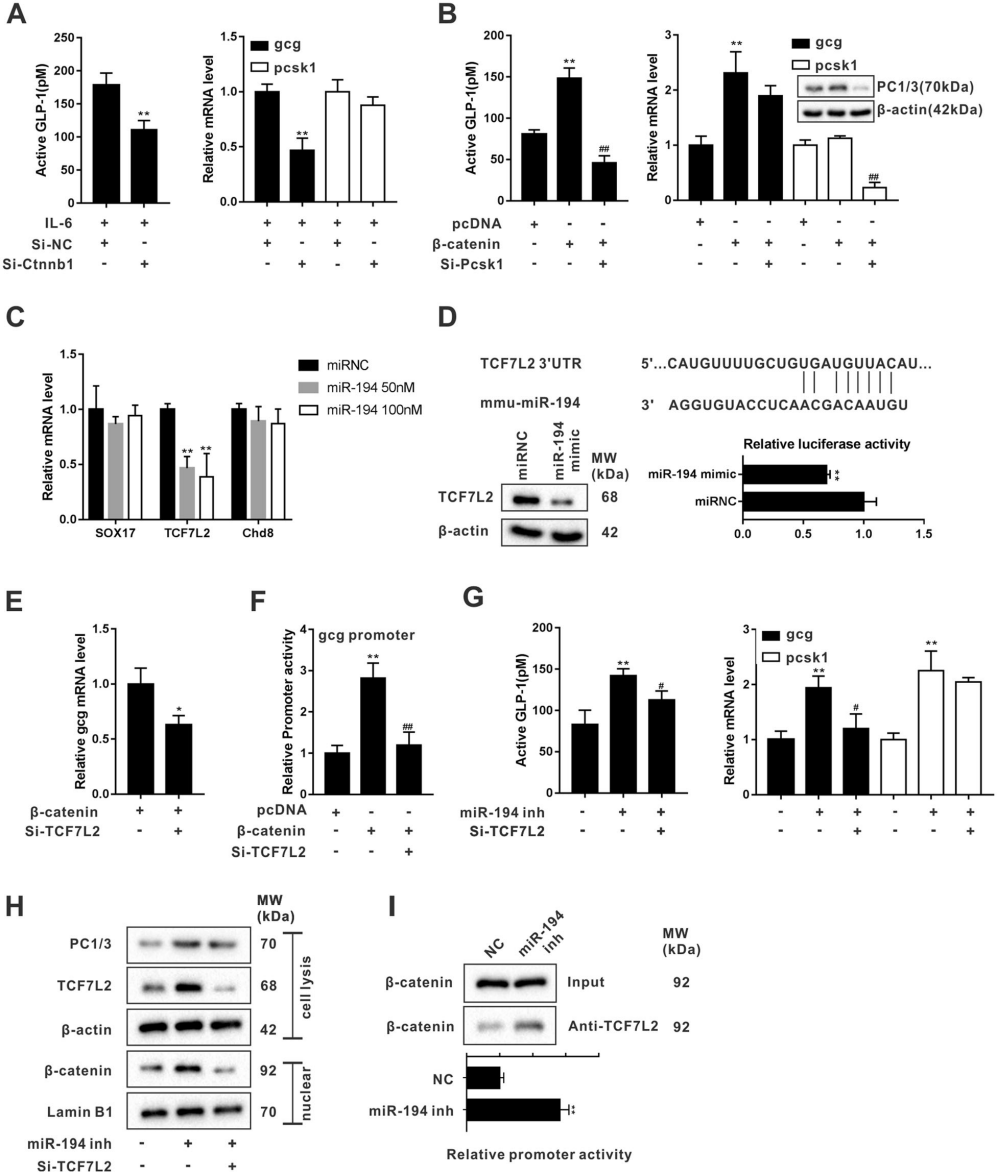
miR-194 reduced GLP-1 synthesis via repressing TCF7L2-mediated *gcg* transcription. **a** The active GLP-1 level and mRNA levels of *gcg* and *pcsk1* were measured in IL-6-treated STC-1 cells which were transfected with si-Ctnnb1 or its negative control (si-NC). **P < 0.01 vs IL-6+si-NC. **b** The active GLP-1 level, mRNA levels of *gcg* and *pcsk1*, and PC1/3 protein level were measured in STC-1 cells which were transfected with pcDNA-β-catenin or its negative control (pcDNA) or pcDNA-β-catenin+si-Pcsk1. **P < 0.01 vs pcDNA. ^##^*P* < 0.01 vs pcDNA-β-catenin. **c** The mRNA levels of sex-determining region Y-box 17 (*SOX17*), transcription factor-7-like 2 (*TCF7L2*), and chromodomain helicase DNA-binding protein 8 (*Chd8*) were determined using qRT-PCR in STC-1 cells which were transfected with miRNC or miR-194 mimic (50 or 100 nM). **P < 0.01 vs miRNC. **d** In STC-1 cells transfected with miRNC or miR-194 mimic, the relative luciferase activity of *TCF7L2* 3’-UTR was measured using the luciferase gene reporter assay and the TCF7L2 protein level was measured by western blot. **P < 0.01 vs miRNC. **e** The *gcg* mRNA level was measure in STC-1 cells which were transfected with pcDNA-β-catenin or pcDNA-β-catenin+si-TCF7L2. *P < 0.05 vs pcDNA-β-catenin. **f** The promoter activity of *gcg* was detected by the luciferase gene reporter assay in STC-1 cells which were transfected with pcDNA or pcDNA-β-catenin or pcDNA-β-catenin+si-TCF7L2. **P < 0.01 vs pcDNA. ^##^*P* < 0.01 vs pcDNA-β-catenin. **g** The active GLP-1 level and mRNA levels of *gcg* and *pcsk1*, and **h** protein levels of PC1/3, TCF7L2, and nuclear β-catenin were measured in STC-1 cells transfected with miR-194 inhibitor or miR-194 inhibitor+ si-TCF7L2. **P < 0.01 vs untreated cells, ^#^*P* < 0.05 vs miR-194 inhibitor. **i** In STC-1 cells transfected with miR-194 inhibitor or its negative control (NC), the endogenous combination between β-catenin and TCF7L2 was detected by co-immunoprecipitation followed by western blot and the promoter activity of *gcg* was detected by luciferase gene reporter assay. **P < 0.01 vs NC.

In IL-6-treated STC-1 cells which were transfected with si-RNA targeting *ctnnb1* (the encoding gene of β-catenin), the silence of β-catenin notably decreased GLP-1 level and *gcg* transcription, while not affecting the *pcsk1* transcription (Fig. 3a), indicating that β-catenin elevating GLP-1 level via facilitating *gcg* transcription. In STC-1 cells cotransfected with pcDNA-β-catenin+si-PCSK1, though the mRNA level of *gcg* was elevated by pcDNA-β-catenin, si-PCSK1 could remove the promoting effect of pcDNA-β-catenin on GLP-1 level (Fig. 3b). The above data hinted that both *gcg* transcription and PC1/3 expression were indispensable for the synthesis of GLP-1.

Firstly, we investigated the mechanism of miR-194 reducing GLP-1 production from the perspective of *gcg* transcription. Considering that miR-194 mimic reduced the nuclear accumulation of β-catenin (Fig. 2j), we speculated that miR-194 inhibited *gcg* transcription via regulating the Wnt/β-catenin signaling pathway. Using bioinformatics software (TargetScan and micro T-CDS), we screened out 191 common target genes of miR-194 (Supplementary Fig. 2a). Then, using the KEGG pathway analysis, we determined 6 potential target genes [calmodulin-dependent protein kinase II gamma(*CAMK2G*), protein phosphatase 3 regulatory subunit B, alpha (*PPP3R1*), sex-determining region Y-box 17 (*SOX17*), transcription factor-7-like 2 (*TCF7L2*), chromodomain helicase DNA-binding protein 8 (*CHD8*), and disheveled associated activator of morphogenesis 1 (*DAAM1*)] of miR-194 which were correlated to the Wnt pathway (Supplementary Fig. 2b). Among them, *CAMK2G*, *PPP3R1*, and *DAAM1* are correlated to non-canonical Wnt signaling, while *SOX17*, *TCF7L2*, and *CHD8* are correlated to canonical Wnt/β-catenin signaling^23–25^. Therefore, we measured the levels of *SOX17*, *TCF7L2*, and *CHD8* in STC-1 cells which were transfected with miR-194 mimic. As shown in Fig. 3c, only the mRNA level of *TCF7L2* exhibited a significantly down-regulation in response to the miR-194 mimic transfection. Subsequently, the luciferase gene reporter assay confirmed that miR-194 mimic suppressed the luciferase activity of *TCF7L2* 3’UTR and the results of western blot showed that miR-194 mimic negatively regulated TCF7L2 expression (Fig. 3d). As depicted in Fig. e–f, the interference of TCF7L2 removed the promoting effect of β-catenin overexpression on *gcg* mRNA level via suppressing the promoter activity of *gcg*, hinting that TCF7L2 was responsible for the regulatory effect of β-catenin on *gcg* transcription. To evaluate whether miR-194 inhibited *gcg* transcription through TCF7L2, STC-1 cells were transfected with miR-194 inhibitor or miR-194 inhibitor+si-TCF7L2. The results of Fig. 3g and h showed that the interference of TCF7L2 reversed the promoting effect of the miR-194 inhibitor on GLP-1 level, gcg transcription, and the protein level of nuclear β-catenin, while not affecting the mRNA and protein levels of PC1/3. In addition, in STC-1 cells transfected with miR-194 inhibitor, a great quantity of β-catenin was detected in complex pulled down by anti-TCF7L2, and the promoter activity of *gcg* was elevated (Fig. 3i). The above data indicating that miR-194 suppressed the activation of the Wnt/β-catenin pathway via negatively regulated TCF7L2 expression, thus inhibited the Wnt/β-catenin pathway-mediated *gcg* transcription.

### miR-194 reduced GLP-1 synthesis via repressing Foxa1-mediated pcsk1 transcription

**Fig. 4:**
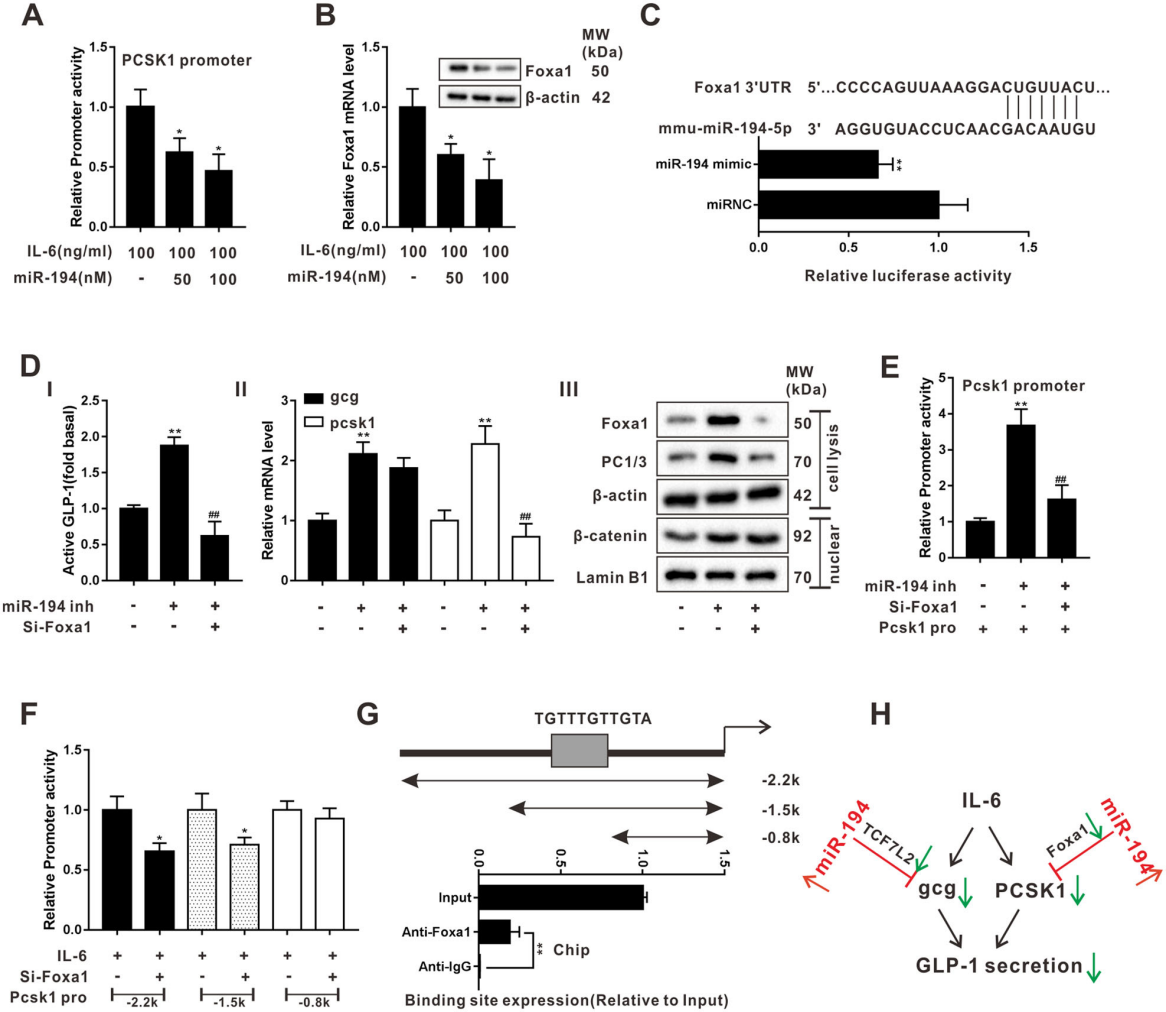
miR-194 reduced GLP-1 synthesis via repressing Foxa1-mediated *pcsk1* transcription. **a** Promoter activity of *pcsk1* and **b** mRNA and protein levels of Foxa1 were detected in IL-6- treated STC-1 cells which were transfected with miR-194 (0 or 50 or 100 nM). *P < 0.05 vs IL-6 treated cells. **c** The relative luciferase activity of *Foxa1* 3’-UTR was measured in the presence of miR-194 mimic or miRNC using the luciferase gene reporter assay. **P < 0.01 vs miRNC. **d** The active GLP-1 level, mRNA levels of *gcg* and *pcsk1*, the protein levels of Foxa1, PC1/3, and nuclear β-catenin, and **e** promoter activity of *pcsk1* were measured in STC-1 cells which were transfected with miR-194 inhibitor or miR-194 inhibitor+si-Foax1. **P < 0.01 vs untreated cells, ^##^*P* < 0.01 vs miR-194 inhibitor. **f** Luciferase activity associated with the region between −2200 to 0 bp, −1500 to 0 bp, and −800 to 0 bp of *pcsk1* promoter in IL-6-treated STC-1 cells which were transfected with si-Foax1. *P < 0.05 vs IL-6-treated cells. **g** Putative Foax1 binding site in the region between −1500 to −800 bp of *pcsk1* promoter. Chromatin immunoprecipitation (CHIP) followed by qRT-PCR was performed to evaluate the binding between Foax1 and the *pcsk1* promoter region between −1500 to −800 bp. The amount of DNA co-immunoprecipitated with each antibody was shown as relative to Input. **P < 0.01 vs Anti-IgG. **h** Mechanism diagram of Figs. 2 to 4.

Following, we evaluated the effect of miR-194 on PC1/3 expression. As shown in Fig. 4a, in response to the overexpression of miR-194, the promoter activity of *pcsk1* was markedly declined in IL-6-treated STC-1 cells. Meanwhile, Foxa1, a transcription factor that contributed to the GLP-1 production^26^, was also down-regulated after miR-194 mimic transfection (Fig. 4b). Importantly, the luciferase activity of *Foxa1* 3’UTR was inhibited in the presence of miR-194 mimic (Fig. 4c). The silence of Foxa1 reversed the miR-194 inhibitor-induced high GLP-1 level, miR-194 inhibitor-up-regulated PC1/3, and miR-194 inhibitor-increased pcsk1 promoter activity, while didn’t significantly change the *gcg* transcription (Fig. 4d and e), suggesting that miR-194 inhibiting *pcsk1* transcription via targeting *Foxa1*, thus stopping PC1/3 from hydrolyzing proglucagon to GLP-1. Whereafter, we constructed the luciferase reporter plasmids containing the *pcsk1* promoter regions between −2200 to 0 bp, −1500 to 0 bp, and −800 to 0 bp. The results of Fig. 4f showed that STC-1 cells transfected with the luciferase reporter plasmids containing the *pcsk1* promoter region between −2200 to 0 bp and −1500 to 0 bp displayed reduced luciferase activity in response to the silence of Foxa1. Furthermore, CHIP assays showed that a great quantity of *pcsk1* promoter region between −1500 to −800 bp was gathered in the complex pulled down by anti-Foxa1 (Fig. 4g), revealing that Foxa1 could bind to *pcsk1* promoter at this region.

### miR-194 mediated the cytotoxicity of PA on an L-like cell line via targeting TCF7L2

**Fig. 5:**
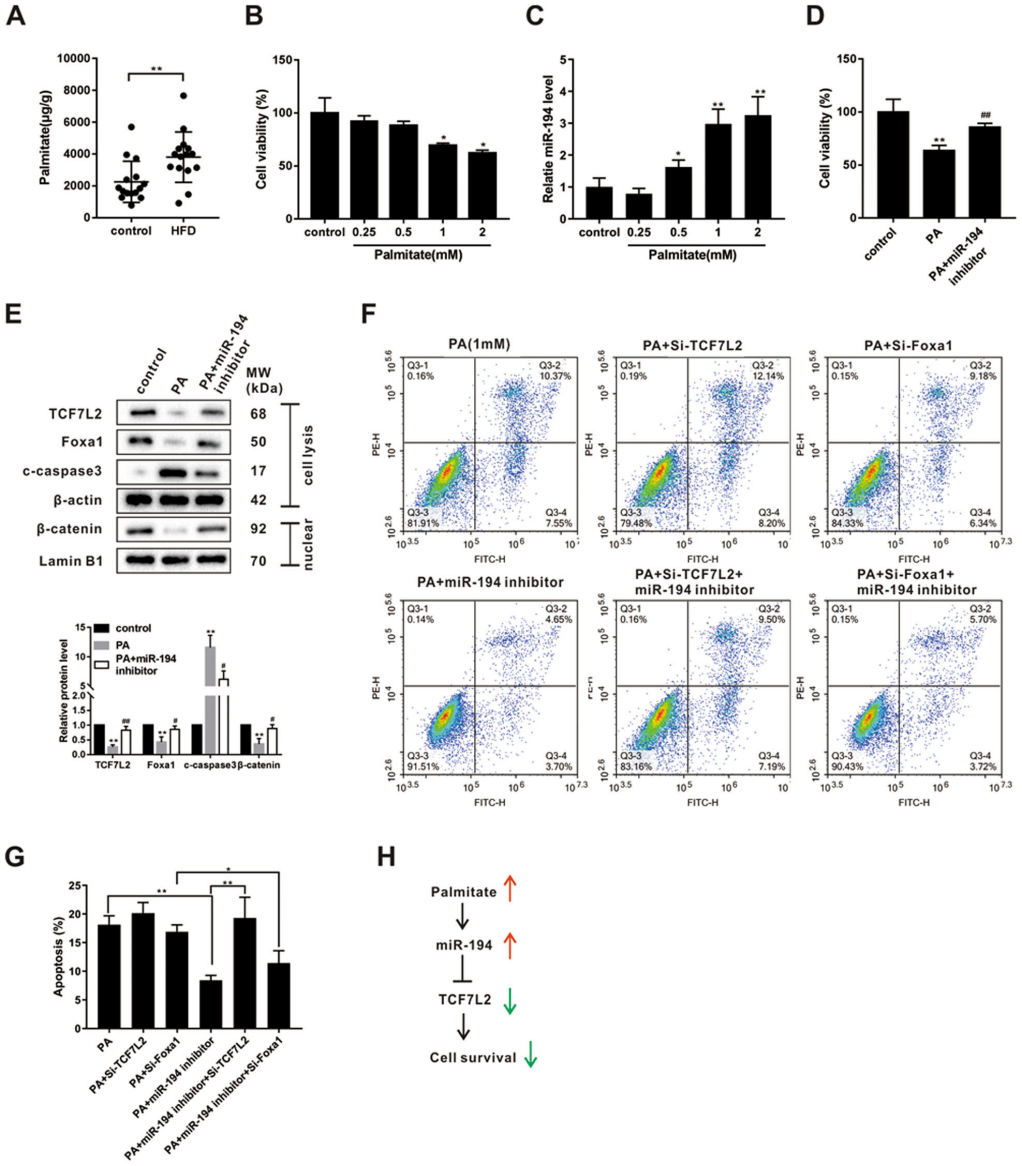
miR-194 mediated the cytotoxicity of PA on an L-like cell line via targeting TCF7L2. **a** The contents of palmitate (PA) from feces of mice in control and HFD groups were detected by chromatography. **P < 0.01. **b**, **c** The STC-1 cells were incubated with different concentrations of PA (0, 0.25, 0.5, 1, and 2 mM) for 24 h. **b** The cell viability was measured by MTT assay. **c** The miR-194 expression was measured by qRT-PCR. *P < 0.05, **P < 0.01 vs control. **d**–**e** The STC-1 cells were transfected with miR-194 inhibitor and incubated with PA (1 mM). **d** Cell viability was measured by MTT assay. **e** The protein levels of TCF7L2, Foxa1, c-caspase 3, and nuclear β-catenin were measured by western blot. **P < 0.01 vs control; ^#^*P* < 0.05, ^##^*P* < 0.01 vs PA. **f**, **g** After transfection of si-TCF7L2 or si-Foxa1 or miR-194 inhibitor or si-TCF7L2 + miR-194 inhibitor or si-Foxa1+miR-194 inhibitor, STC-1 cells were incubated with PA (1 mM) for 24 h. **f** Cell apoptosis was measured by flow cytometry and the quantified results were shown in (**g**). *P < 0.05, **P < 0.01. **h** Mechanism diagram of Fig. 5.

The PA level was significantly elevated in the feces of HFD-fed mice (Fig. 5a). The high concentration of PA (>0.5 mM) reduced cell viability and up-regulated miR-194 expression in the STC-1 cells (Fig. 5b and c). Since the miR-194 target genes, *TCF7L2* and *Foxa1*, were proved to be involved in the regulation of cell growth^27,28^, we speculated that miR-194 may play a role in PA-induced cytotoxicity via regulating TCF7L2 and Foxa1. Notably, the interference of miR-194 reversed the inhibitory effect of PA on cell viability (Fig. 5d) and the protein levels of Foxa1, TCF7L2, and nuclear β-catenin, and removed the promoting effect of PA on c-caspase 3 protein level (Fig. 5e). Then, as shown in Figure 5f and g, in PA-treated STC-1 cells, the silence of miR-194 reduced PA-induced cell apoptosis, the si-TCF7L2, rather than si-Foxa1, abrogated the effect of miR-194 inhibitor, hinting that miR-194 participated in PA-induced apoptosis via targeting *TCF7L2*.

### miR-194 knockdown induced GLP-1 synthesis in HFD-induced obese mice

**Fig. 6:**
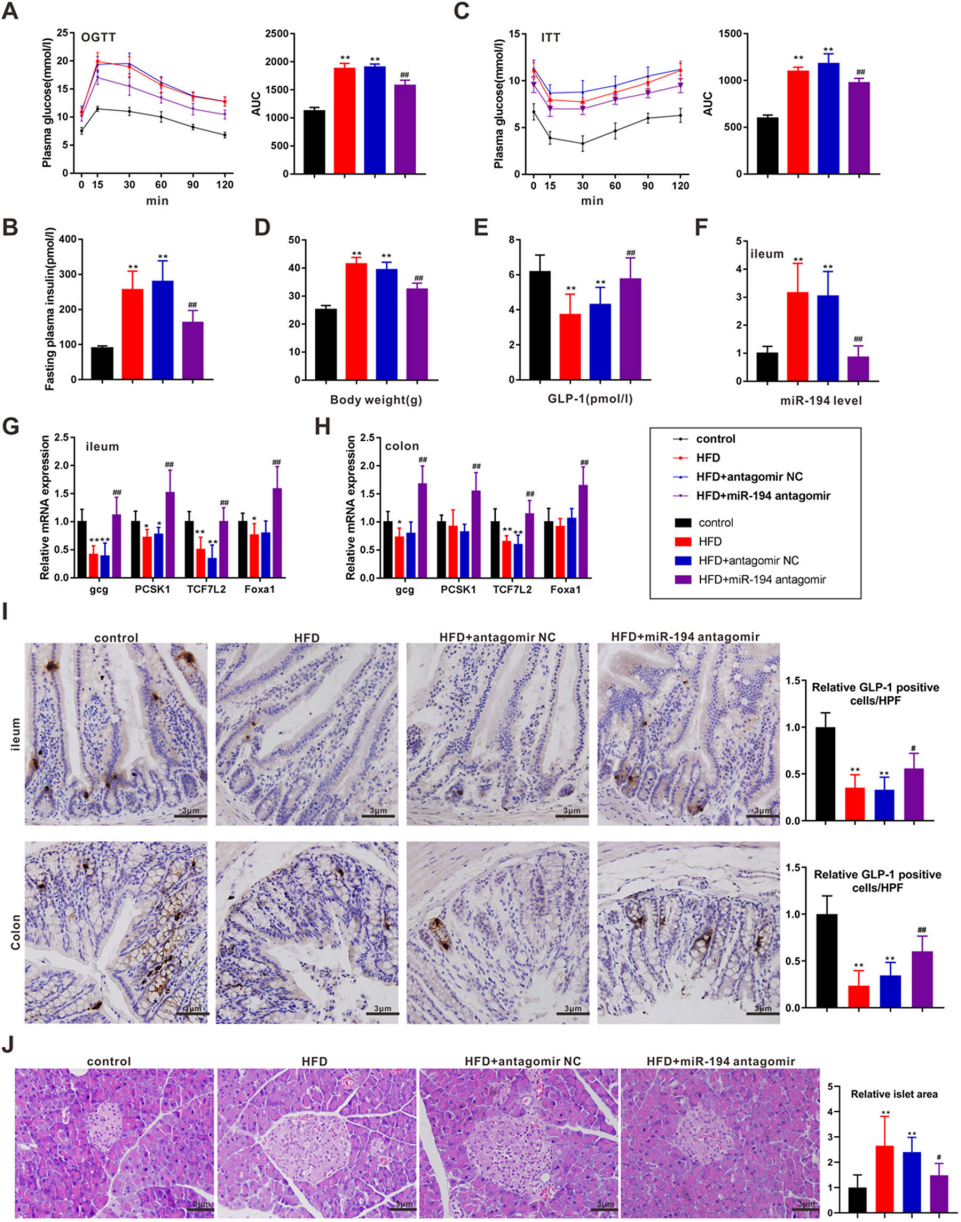
miR-194 knockdown induced GLP-1 synthesis in HFD-induced obese mice. Male C57BL/6 mice were divided into control (*n* = 6), HFD (*n* = 6), HFD + antagomir NC (*n* = 6), and HFD + miR-194 antagomir (*n* = 6) groups. **a** Oral glucose tolerance test (OGTT) was performed and the area of OGTT curve was measured (AUC). **b** The plasma insulin level was determined by ELISA. **c** The insulin tolerance test (ITT) was performed and the area of the ITT curve was measured (AUC). **d** Body weight of mice in each group. **e** The plasma level of active GLP-1 was assayed by ELISA. **f** The miR-194 expression in ileum tissues was measured by qRT-PCR. The mRNA levels of *gcg*, *pcsk1*, *TCF7L2*, and *Foxa1* were determined in **g** ileum and **h** colon tissues of mice. **i** Representative immunohistochemical staining for GLP-1 in ileum and colon tissues (scale bar=3 μm) and quantified results were expressed as the mean numbers of GLP-1-positive cells in HPFs. **j** Representative H&E staining performed on pancreatic tissues (scale bar = 3 μm). *P < 0.05, **P < 0.01 vs control; ^#^*P* < 0.05, ^##^*P* < 0.01 vs HFD + antagomir NC.

To verify our in vitro findings in vivo, miR-194 was silenced in ileum tissues of HFD-induced obese mice utilizing intracolonic enemas of miR-194 antagomir (Fig. 6f). As depicted in Fig. 6a, b and c, oral glucose tolerance and insulin tolerance induced by HFD were relieved in miR-194 antagomir-treated mice compared with antagomir NC-treated mice. Meanwhile, miR-194 knockdown declined the body weight, islet area, and plasma levels of TG, TC and IL-6 in HFD-fed mice (Fig. 6d, j, and Table 3). In addition, the levels of GLP-1 in plasma, ileum, and colon were up-regulated by miR-194 antagomir (Fig. 6e and i). The high mRNA levels of *gcg*, *pcsk1*, *TCF7L2*, and *Foxa1* in ileum and colon tissues of miR-194 antagomir-treated mice (Fig. 6g and h) displayed that miR-194 knockdown promoted GLP-1 synthesis via up-regulating TCF7L2/*gcg* and Foxa1/*pcsk1*.

**Table 3:** miR-194-5p antagomir on biochemical characteristics.

Group	TG (mM)	TC (mM)	IL-6 (pg/ml)
Control (*n* = 6)	1.07 ± 0.31	3.09 ± 0.57	112.58 ± 13.79
HFD (*n* = 6)	1.47 ± 0.25^*^	5.49 ± 0.79^**^	195.22 ± 19.42^**^
HFD + antagomir-NC (*n* = 6)	1.64 ± 0.34^*^	5.64 ± 0.87^**^	213.74 ± 18.08^**^
HFD + miR-194 antagomir (*n* = 6)	1.21 ± 0.20^#^	4.16 ± 0.83^##^	166.11 ± 26.60^##^

TC, total cholesterol; TG, triglyceride; HFD, high-fat diet; antagomir-NC, the negative control of miR-194 antagomir. *P < 0.05, **P < 0.01 vs control; ^#^*P* < 0.05, ^##^*P* < 0.01 vs HFD + antagomir-NC.

## Discussion

The major finding of the present study is that miR-194 functions to regulate GLP-1 synthesis in L cells. This conclusion was supported by the following distinct observations: (1) a negative correlation existed between plasma GLP-1 level and ileac miR-194 expression in HFD-induced obese mice; (2) miR-194 overexpression reduced GLP-1 level in IL-6-treated STC-1 cells via repressing transcriptions of *gcg* and *pcsk1*; (3) miR-194 knockdown reduced PA-induced cell apoptosis in STC-1 cells; (4) miR-194 knockdown relieved the metabolic symptoms caused by GLP-1 deficiency in HFD-induced obese mice.

MiRNAs have been reported to be involved in several metabolic diseases via negatively regulate gene expression at the post-transcriptional level by binding to the mRNAs^29^. For example, in type 2 diabetes mellitus (T2DM), miR-125a-5p was been reported to ameliorate hepatic glycolipid metabolism disorder through targeting signal transducer and activator of transcription 3 (STAT3)^30^. Meanwhile, miR-148a proved to be a negative regulator of low-density lipoprotein receptor (LDLR) through targeting sterol regulatory element-binding protein 1^31^. Nevertheless, the studies focused on the role of miRNA in the synthesis of GLP-1, a pivotal regulator of glucose and lipid metabolism^29^, are still lacked. In the present study, an apparent up-regulation of miR-194 was observed in the obese mice model (Fig. 1c). The overexpression of miR-194 abolished the IL-6-induced high levels of GLP-1, *gcg* mRNA, and PC1/3 protein (Fig. 2e–g). The subsequent experiments clarified that the miR-194 suppressed *gcg* mRNA and PC1/3 protein via directly targeting *TCF7L2* and *Foxa1*, respectively (Figs. 3 and 4). Our data thus firstly provide evidence that miR-194 could modulate GLP-1 production via transcription and protein levels.

β-catenin is a key transcriptional coactivator of the canonical Wnt pathway, its accumulation in nuclear marks the activation of Wnt/β-catenin signaling pathway and enhanced the transcription of Wnt target genes^32^. Ni et al.^21^ reported that *gcg* was among the target genes of the Wnt/β-catenin signaling pathway in GLUTag cells. Indeed, in our study, the high level of GLP-1 induced by IL-6 was associated with the up-regulation of nuclear β-catenin (Fig. 2b and g), and the silence of β-catenin suppressed the *gcg* mRNA level and reduced GLP-1 level (Fig. 3a). The previous study has proved that miR-194 could inactive Wnt/β-catenin signaling pathway via suppressing the nuclear accumulation of β-catenin, thus inhibiting cell invasion in hepatocellular carcinoma cells^33^. Consistent with this, a decreased nuclear β-catenin expression was observed after miR-194 overexpression (Fig. 2j). What’s more, our study also found that TCF7L2, a Wnt-transcription factor^34^, was a direct target of miR-194 (Fig. 3d). As reported, in the absence of Wnt stimulus, cytoplasmic β-catenin combined with glycogen synthase kinase-3β and is then phosphorylated and ubiquitinated^32^. When the Wnt signaling is activated, binding of Wnt to its receptors disrupts the β-catenin destruction complex and promotes the nuclear translocation of β-catenin. Then, nuclear β-catenin interacts with the T-cell factor (such as TC7F2) to transcriptionally regulate gene expression^35^. Therefore, we speculated that miR-194 regulated Wnt/β-catenin signaling pathway-mediated *gcg* transcription via targeting *TCF7L2*. The subsequent experiments displayed that the silence of TCF7L2 removed the promoting effect of β-catenin on *gcg* promoter activity (Fig. 3e and f). In addition, in miR-194 silenced STC-1 cells, the combination between TC7F2 and β-catenin was promoted, resulted in an elevated *gcg* promoter activity (Fig. 3i). These observations collectively showed that TC7F2 was responsible for the regulatory effect of the Wnt/β-catenin signaling pathway on *gcg* transcription and TC7F2 was a control point in the regulatory effect of miR-194 on *gcg* transcription.

Except for *gcg*, PC1/3 is also an indispensable participator during the progress of GLP-1 production^36^. Foxa1 is a winged-helix transcription factor expressed in the definitive endoderm during embryogenesis and has been proved to be a contributor to the L cell differentiation^26^. Baraille et al.^37^ found that the increased expression of Foxa1 led to an increase of GLP-1 plasma levels in mice while the mechanism remained unclear. In our study, we clarified that Foxa1 directly bound to the −1500 to −800 bp *pcsk1* promoter activity, thus enhancing *pcsk1* transcription (Fig. 4). Therefore, our study firstly illustrated the internal mechanism of the regulatory effect of Foxa1 on GLP-1 production and providing a new intervention target for GLP-1 synthesis.

It was previously reported that lipotoxicity, induced by PA, was able to reduce GLP-1 secretion and caused endoplasmic reticulum stress and apoptosis in L cell lines^38^. In our in vitro experiment, we also observed that treatment with high PA concentrations (1 mM for 24 h) induced cell apoptosis of STC-1 cells (Fig. 5b). Of note, the miR-194 knockdown removed the proapoptosis effect of PA through targeting *TCF7L2* (Fig. 5f and g). Therefore, we summarized that, except for directly regulated GLP-1 synthesis via targeting *TCF7L2* and *Foxa1*, miR-194 could also indirectly repressed GLP-1 production by promoting PA-induced apoptosis of L cells.

In conclusion, the current study indicated that miR-194 suppressed GLP-1 synthesis via reducing TCF7L2-mediated *gcg* transcription and Foax1-mediated *pcsk1* transcription. Additionally, miR-194 was also responsible for the PA-induced cell apoptosis in L cells. Our study firstly expounded the regulatory effect of miR-194 on GLP-1 synthesis in intestinal L cells, providing a novel target of treating metabolic syndrome caused by obesity.

## Supplementary information

Supplementary Figure Legends

Supplemental Figure 1

Supplemental Figure 2
