# Supplementary material for: MicroRNA-194: a novel regulator of glucagon-like peptide-1 synthesis in intestinal L cells
Source: Cell Death Dis. 2021 Jan 21;12(1):113. doi: 10.1038/s41419-020-03366-0 (PMC7820456; doi:10.1038/s41419-020-03366-0)
Supplement: Supplementary file 1 — Supplementary Figure Legends [file 41419_2020_3366_MOESM1_ESM.docx]

**Supplementary Figure 1** Male C57BL/6 mice were fed with a normal chow diet (control group, *n*=15) or high-fat diet (HFD group, *n*=15) for 12 weeks. (A) Body weights of mice in control and HFD groups. (B) Biochemical characteristics of mice. TC=total cholesterol; TG=triglyceride. (C) Representative H&E staining performed on adipose tissues (scale bar=100 μm) and quantified results. (D) Representative H&E staining performed on pancreatic tissues. ***P*<0.01 vs control.

**Supplementary Figure 2** (A) The potential target genes of miR-194 were predicted by TargetScan and Micro T-CDS. (B) The KEGG pathway analysis.
