# Supplementary figures and images for: MicroRNA-194: a novel regulator of glucagon-like peptide-1 synthesis in intestinal L cells
Source: Cell Death Dis. 2021 Jan 21;12(1):113. doi: 10.1038/s41419-020-03366-0 (PMC7820456; doi:10.1038/s41419-020-03366-0)

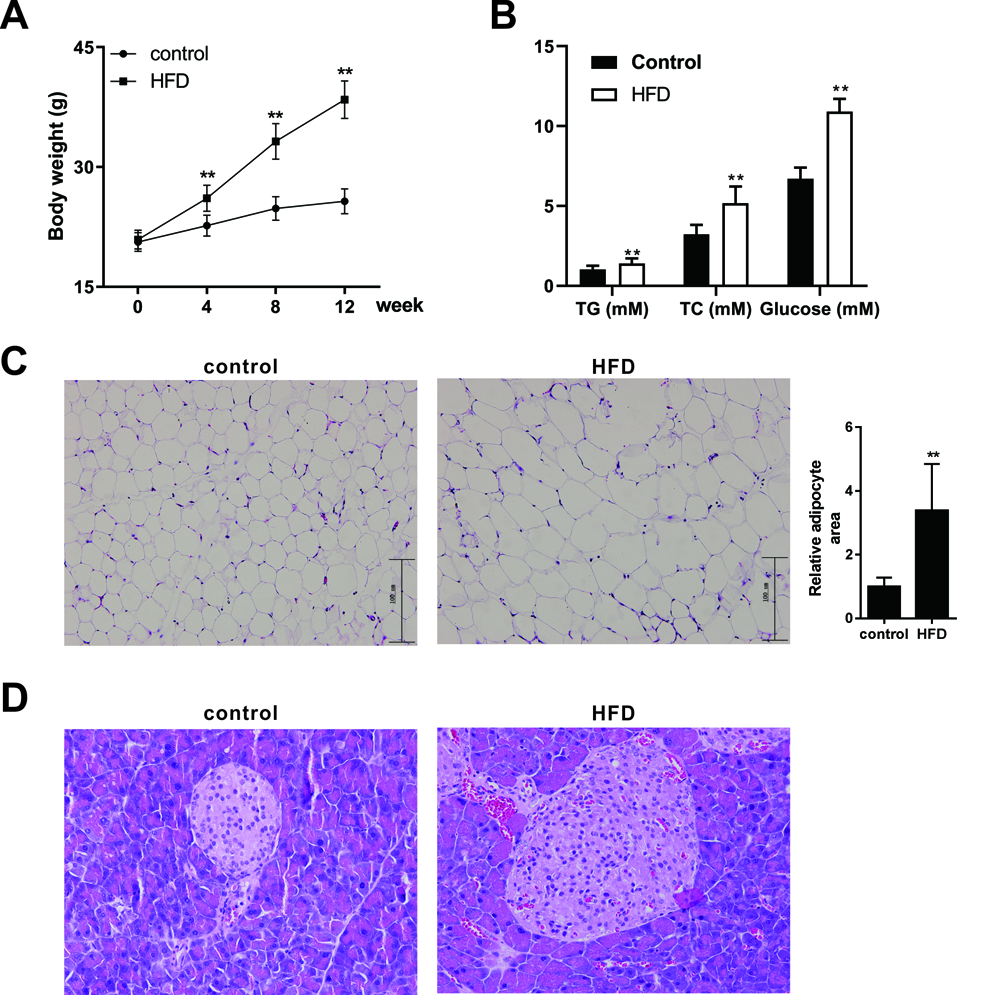

Supplement: Supplementary file 2 — Supplemental Figure 1 [file 41419_2020_3366_MOESM2_ESM.tif]

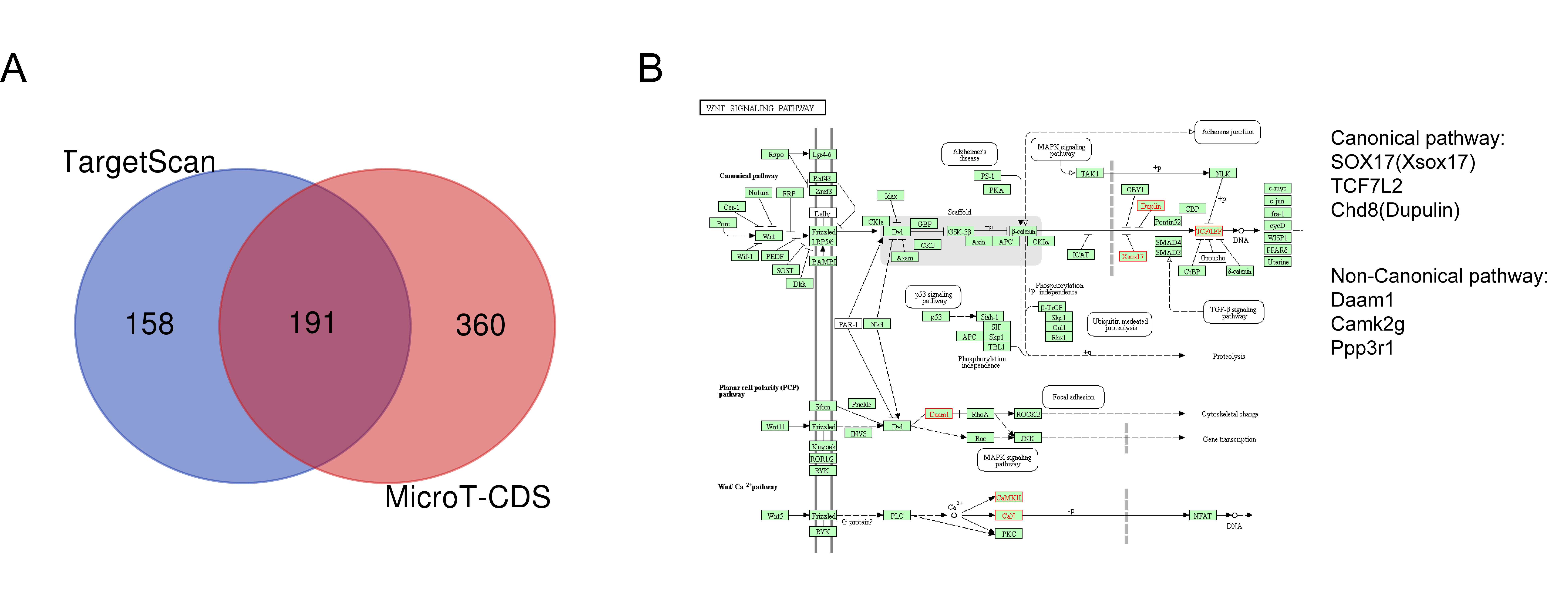

Supplement: Supplementary file 3 — Supplemental Figure 2 [file 41419_2020_3366_MOESM3_ESM.tif]
